# Evaluating Cognitive Function and Brain Activity Patterns via Blood Oxygen Level-Dependent Transformer in N-Back Working Memory Tasks

**DOI:** 10.3390/brainsci15030277

**Published:** 2025-03-05

**Authors:** Zhenming Zhang, Yaojing Chen, Aidong Men, Zhuqing Jiang

**Affiliations:** 1 School of Artificial Intelligence, Beijing University of Posts and Telecommunications, Beijing 100876, China; zhenmingzhang@bupt.edu.cn (Z.Z.); menad@bupt.edu.cn (A.M.); 2 State Key Laboratory of Cognitive Neuroscience and Learning, Beijing Normal University, Beijing 100875, China; chenyaojing@bnu.edu.cn; 3 Beijing Key Laboratory of Network System and Network Culture, Beijing University of Posts and Telecommunications, Beijing 100876, China

**Keywords:** working memory, N-back, BolT, cognition process, brain activation pattern

## Abstract

(1) Background: Working memory, which involves temporary storage, information processing, and regulating attention resources, is a fundamental cognitive process and constitutes a significant component of neuroscience research. This study aimed to evaluate brain activation patterns by analyzing functional magnetic resonance imaging (fMRI) time-series data collected during a designed N-back working memory task with varying cognitive demands. (2) Methods: We utilized a novel transformer model, blood oxygen level-dependent transformer (BolT), to extract the activation level features of brain regions in the cognitive process, thereby obtaining the influence weights of regions of interest (ROIs) on the corresponding tasks. (3) Results: Compared with previous studies, our work reached similar conclusions in major brain region performance and provides a more precise analysis for identifying brain activation patterns. For each type of working memory task, we selected the top 5 percent of the most influential ROIs and conducted a comprehensive analysis and discussion. Additionally, we explored the effect of prior knowledge conditions on the performance of different tasks in the same period and the same tasks at different times. (4) Conclusions: The comparison results reflect the brain’s adaptive strategies and dependencies in coping with different levels of cognitive demands and the stability optimization of the brain’s cognitive processing. This study introduces innovative methodologies for understanding brain function and cognitive processes, highlighting the potential of transformer in cognitive neuroscience. Its findings offer new insights into brain activity patterns associated with working memory, contributing to the broader landscape of neuroscience research.

## 1. Introduction

The brain’s abilities involved in acquiring, processing, storing, and retrieving information are known as cognitive functions. Working memory, considered the foundation of cognitive functions, is an important experimental paradigm in neuroscience for assessing the temporary storage and manipulation of information in the brain [1,2]. These tasks typically involve presenting participants with a sequence of stimuli, such as numbers, letters, or shapes, and requiring them to retain and manipulate this information over a brief duration. Initially proposed by Kirchner in 1958 [3], the N-back task is one of the most widely employed paradigms in cognitive neuroscience research for investigating working memory. Due to its demand for both the retention and manipulation of cognitive information, this task has been extensively utilized in neuroimaging studies. In the N-back task, participants are presented with a series of stimuli and are instructed to respond by button-press when the current stimulus matches the one presented *N* steps back in the sequence. This task is administered with various values of *N*, and increasing *N* levels heighten the task’s difficulty due to the heightened complexity of cognitive load [4,5]. A baseline condition (0-back) is included where participants must respond if a stimulus equals a predefined item.

Functional magnetic resonance imaging (fMRI) explores complex cognitive processes in the human brain [6,7,8] by measuring blood oxygen level-dependent (BOLD) responses that indicate changes in metabolic demand following neural activity [9,10]. Task-based fMRI associates stimulus or task variables with brain responses [11,12,13] to identify co-activated brain regions [14] and indicate functional connectivity [15]. Neuroimaging studies, particularly those employing fMRI, have advanced the understanding of the activated brain regions involved in N-back tasks. For instance, Owen et al. [16] conducted the first meta-analysis of fMRI studies on the N-back task in adults, identifying key regions such as the prefrontal and parietal cortices. Similarly, Rottschy et al. [17] confirmed the involvement of an extensive frontoparietal network in healthy adults, and Wang et al. [18] further demonstrated that regions like the middle frontal gyrus, inferior parietal lobule, and thalamus are consistently activated across different memory loads. These findings have provided valuable insights into the neural mechanisms underlying working memory tasks like the N-back.

Conventionally, statistical methods and traditional machine learning have been utilized to process fMRI data, aiming to estimate spatiotemporal patterns associated with cognition and brain diseases. Feature extraction is commonly employed to reduce dimensionality and mitigate nuisance variability [19,20]. Functional connectivity features are typically expressed as temporal correlations of BOLD responses across distinct brain regions. Methods such as support vector machines or logistic regression are then applied to classify relational variables [21,22,23,24]. While these approaches have proven valuable, they are limited in their ability to capture complex and nonlinear relationships within high-dimensional fMRI data. Recently, many studies have instead applied deep learning due to its capacity to capture intricate patterns in high-dimensional data, thus investigating the nonlinear relationship between brain dynamics and human cognition/behaviors [25,26,27]. Various successful deep learning models have emerged in the literature, leveraging convolutional [28], graph [29], or recurrent architecture [30,31] to process functional connectivity features. Moreover, several recent studies have opted for transformer models [32,33,34] to build a classifier directly on BOLD responses, enabling a more direct assessment of high-order interactions in fMRI data.

In this paper, we aimed to evaluate the brain activity patterns associated with cognitive function using deep learning techniques. To detect these cerebral activation patterns, we designed a working memory N-back task with three levels of cognitive demand. While brain cognitive patterns in N-back tasks have been extensively studied, recent research has demonstrated the precision and sensitivity of deep learning techniques in describing activated brain regions. This introduces innovative perspectives and methodologies for neuroscience research. In our study, we employed the blood oxygen level-dependent transformer (BolT) model to capture brain activity patterns across varying levels of cognitive task difficulty [35]. The experimental results are consistent with prior research and offer a more precise and detailed analysis of activated brain regions compared to traditional statistical methods. We also investigated the brain’s performance on (1) the same task under different prior knowledge conditions and (2) task classification at different time stages.

## 2. Methods and Materials

### 2.1. Datasets Description

Participants were recruited from the Beijing Aging Brain Rejuvenation Initiative (BABRI) study [36], an ongoing longitudinal investigation focusing on brain health and cognitive decline in elderly individuals residing in the community. Inclusion criteria for participants in this report were as follows: (1) native Chinese speakers aged over 50 years of age without dementia and possessing normal daily living abilities; (2) no history of brain tumors, neurological or psychiatric disorders, or substance addiction; (3) not presenting with conditions known to affect cerebral function, including alcoholism, current depression, Parkinson’s disease, or epilepsy; and (4) no contraindications to magnetic resonance imaging (MRI). Finally, 255 individuals met these criteria and were included in the study.

All participants underwent scanning with a Siemens Trio 3T scanner (Siemens Healthineers, Erlangen, Germany) located at the Imaging Center for Brain Research at Beijing Normal University. Participants were laid supine, with their heads securely immobilized, using straps and foam pads to minimize head movement. High-resolution T1-weighted sagittal 3D magnetization prepared rapid gradient echo sequences were obtained, covering the entire brain (176 sagittal slices; repetition time = 1900 ms; echo time = 3.44 ms; slice thickness = 1 mm; flip angle = 9°; inversion time = 900 ms; field of view = 256×256 ${\mathrm{mm}}^{2}$; acquisition matrix = 256 × 256).

### 2.2. N-Back Task Design

The N-back task in this study was performed with visual stimuli consisting of selected numbers. The task involved presenting a series of visual stimuli to participants in a predetermined sequence, preceded by a 10-second guidance before each appearance. The task comprised 9 blocks, divided into 3 blocks per N-back condition ranging from easy to difficult (0-back, 1-back, and 2-back). In total, 180 stimuli (20 per block) were presented with different targets occurring thrice across the three conditions.

**Figure 1:**
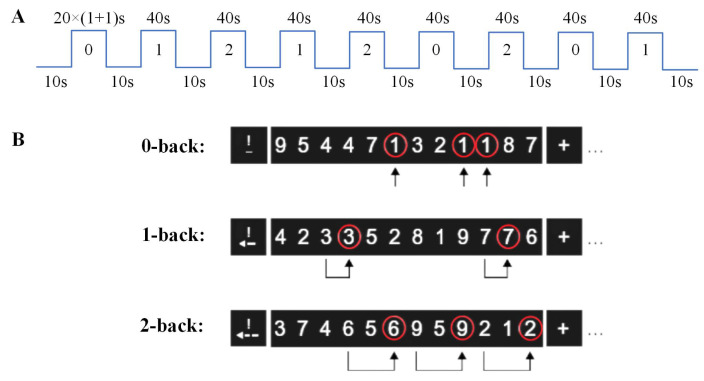
(**A**) The working memory task adopts the numerical N-back paradigm, with three levels from easy to difficult: 0-back, 1-back, and 2-back. The three levels appear pseudo-randomly 3 times, with 10 seconds of instruction before each appearance. The task comprises a total of 9 blocks, with each block containing 20 trials. Among these, only 6 trials require a correct button response. (**B**) Illustration of the N-back working memory task paradigm with three levels of difficulty.

Participants were instructed to press the button with their right hand under three conditions: when the displayed number matched a predefined target number (0-back), when consecutive identical numbers appeared (1-back), or when identical numbers with the same interval were presented (2-back). Notably, we also included target trials where subjects refrained from responding. This was done to ensure equal and adequate trials for all conditions and participants. See Figure 1 for an illustration of the paradigm.

### 2.3. Data Preprocessing

Data preprocessing was conducted using statistical parametric mapping (SPM12) software. Registration with higher degrees of freedom and segmentation operations were applied to analyze 3D T1-weighted scans. Firstly, we utilized slice timing to mitigate potential confounds caused by temporal differences in slice acquisition within each volume of fMRI data. Next, we realigned fMRI images to correct distortion from head motion and co-registrate functional images with T1-weighted anatomical images. Finally, we normalized images into Montreal Neurological Institute (MNI) space and apply spatial smoothing with a Gaussian kernel of 6 ${\mathrm{mm}}^{3}$ to reduce spatial noise.

**Figure 2:**
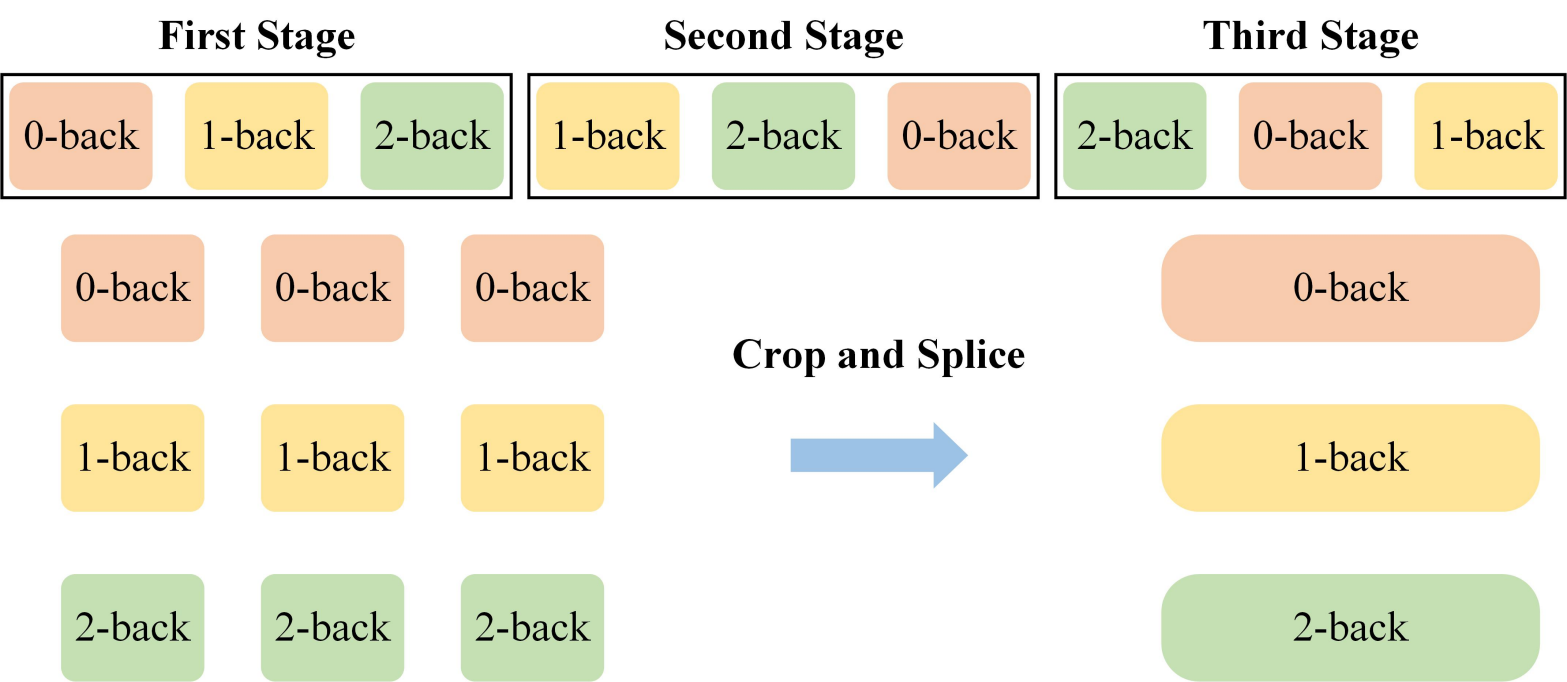
The entire task process is divided into three stages, with the same task blocks from each stage being cropped and spliced.

After that, we segmented the data according to the time series for the 0-back, 1-back, and 2-back tasks, and classification experiments were designed for each task. However, one block only contains 20 trials, which might not provide sufficient information for feature extraction by the classifier and probably causes overfitting problems. To address this, we cropped and spliced three blocks of the same task to increase the number of sampling points within a block. This approach expanded the operating space for the model to extract features and laid the groundwork for determining the most suitable hyperparameters for the model. Figure 2 shows a schematic diagram of block processing.

### 2.4. Analysis Techniques

In our study, we adopted a novel transformer model, BolT [35], and designed a classification task to analyze the fMRI data. BolT has gained prominence in the analysis of fMRI data due to its capability to capture temporal dynamics directly from fMRI time series. In contrast to traditional methods that primarily rely on clustering and probabilistic approaches to identify the most relevant brain regions, BolT offers a significant advantage by providing importance weights for all brain regions, thereby ensuring a more comprehensive analysis. BolT has been shown to outperform other machine learning and deep learning techniques, achieving superior accuracy and sensitivity. This enhancement facilitates a more precise and nuanced understanding of the brain’s responses to cognitive tasks. Although BolT has not been extensively evaluated for task-specific regions or fine-grained classification tasks, we focused on the more specific N-back working memory task, utilizing BolT to investigate the activated brain regions during the task. BolT leverages the Transformer architecture, separating spatial and temporal attention units and focusing on local representations, with the fused window multi-head self-attention (FW-MSA) module. The FW-MSA module calculates local attention within adjacent time windows, significantly enhancing the capture of subtle changes in the dynamics of brain activations while maintaining the linear scalability of the fMRI time series [37].

**Figure 3:**
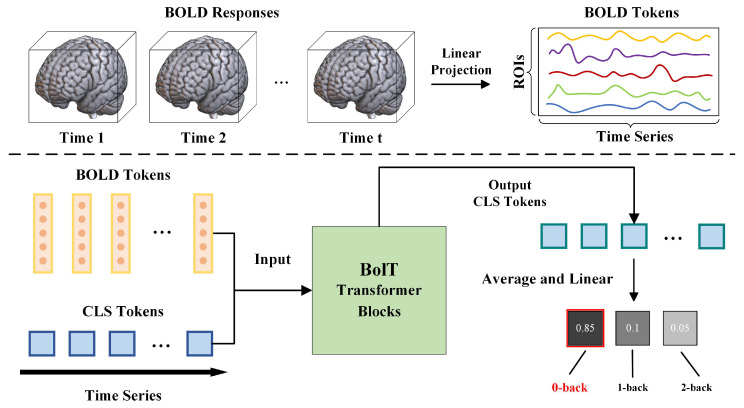
Classification task process overview. The BOLD responses are extracted from the fMRI time series and projected to the corresponding brain region according to the external brain atlas to obtain BOLD tokens. Each BOLD token encodes the ROI responses for the corresponding period. Cascade transformer blocks process these BOLD tokens across a series of overlapping time windows in the time series. A separate learnable CLS token is introduced into the transformer blocks for each time window. Both the BOLD tokens and the CLS tokens serve as inputs to the transformer blocks, facilitating the extraction of latent representations. Finally, the output CLS tokens are averaged and passed through a linear layer to yield the final classification results.

We utilized the external Schaefer brain atlas that comprises 400 regions labelled across seven intrinsic connectivity networks [38] to extract the regional BOLD responses and map the four-dimensional fMRI time-series data to the corresponding ROIs. The time series of ROI was obtained by averaging the responses across voxels and aligned with the MNI template. The task of the model in the classification was to map these regional BOLD responses to the class label. An overview of the classification task process is shown in Figure 3.

### 2.5. Implementation Details

The experiments were conducted in PyTorch 1.12.1 on an NVIDIA RTX 2080 Ti GPU (NVIDIA Corporation, Santa Clara, CA, USA). Modelling utilized a five-fold cross-validation procedure to evaluate the model’s performance on different subsets and enhance its generalization ability. FMRI time series were dynamically sampled at randomly generated start positions to enable the model to capture patterns and correlations better, thus improving learning efficiency. The time series were standardized to maintain consistent value ranges, optimizing convergence speed and stability.

Hyperparameter selection was based on performance in the initial validation set. Parameters demonstrating near-optimal performance across all datasets and atlases were selected. The selected parameters included learning rate $\in (2\times 10^{-6},4\times 10^{-4})$, 20 epochs, and mini-batch size ∈(8,32). The training was performed via the Adam optimizer. BolT was trained to minimize the following loss: $L=L_{CE}+\lambda \cdot L_{CLS}$ where $L_{CE}$ is cross-entropy loss, and λ=0.1 is the regularization coefficient for CLS loss set via cross-validation.

Based on the number of subjects, BolT was trained for 20 epochs with a batch size of 16. A hidden dimensionality of 400 and 36 attention heads with 20 dimensions per head was prescribed. A dropout rate of 0.1 was used in both FW-MSA and MLP layers. For the FW-MSA architecture, given a desired dynamic length *D*, receptive field *R*, and window size *W*, stride *s*, fringe length *L*, and the number of layers *N* were set proportionately as follows:

(1) $$\begin{array}{cc} \; & s=W\alpha \end{array}$$

(2) $$\begin{array}{cc} \; & L=2\times (1-\alpha )W \end{array}$$

(3) $$\begin{array}{cc} \; & D>R=W+(N-1)\times s \end{array}$$

where α∈(0,1) is the stride coefficient, which is a preset proportionality constant. Dynamic length represents the application range of the FW-MSA structure and determines the range within which the model dynamically samples the time series. A larger dynamic length accommodates more transformer blocks for feature extraction.

Window size refers to the size of the MSA module. Due to the limitation of the length of the time series, balancing the relationship between length and quantity requires more consideration (a larger length means fewer modules). Given the short time series, prioritizing the amount of modules becomes more significant. The receptive field is formed by adding fringe blocks on both sides of the fused window, effectively increasing the effective number of layers. The receptive field needs to cover the range of dynamic length as much as possible to accommodate more transformer blocks for feature extraction. The following values were selected for the hyperparameters: D=50;N=12;W=5;α=0.8. The specific hyperparameter adjustment process is shown in Table 1.

## 3. Results

### 3.1. Importance Weight Characteristics of Brain Regions

**Figure 4:**
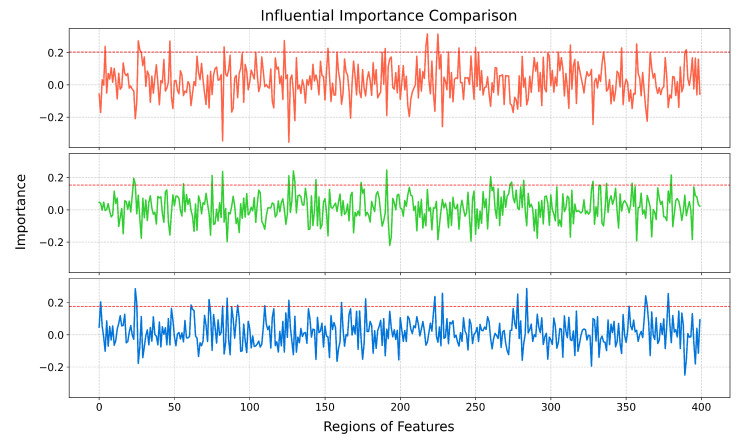
The importance weights for the ROIs of each N-back task corresponding to the Schaefer brain atlas in the working memory task. The horizontal axis represents the different brain regions of the Schaefer brain atlas. The brain regions above the red line are considered to be the regions that contribute the most to the task.

After the classification task achieved convincing accuracy, we evaluated the impact of the BOLD tokens by computing the gradient-weighted attention maps [39] and the correlation scores between tokens to determine their importance for the classification task. The importance weights for the ROIs of each N-back task corresponding to the Schaefer brain atlas in the working memory task are shown in Figure 4.

We used the landmark time point to identify the brain regions crucial for the detection task. Five tokens were extracted for each subject to represent responses across ROIs. Subsequently, a logistic regression model was trained to correlate the tokens at landmark time points with their respective output classes. The model weights signify the contribution of each ROI to the classification decision. We analyzed the importance weights of influential ROIs to determine their significance in each task and elucidate the neural correlates underlying the processing demands of these cognitive tasks.

**Figure 5:**
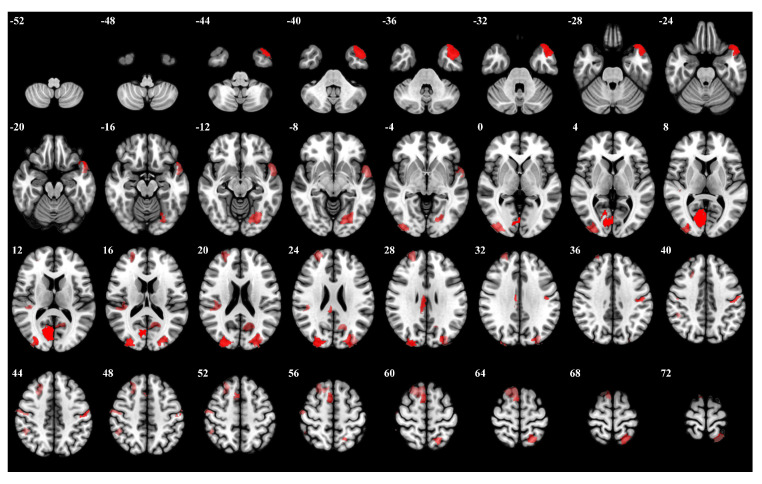
The top 5 percent of the most influential ROIs for 0-back, with higher opacity indicating higher influence weights.

In the 0-back task, participants were instructed to respond to a specific target number stimulus. The right hemisphere visual cortex, particularly regions Right Hemisphere Visual 19 and Right Hemisphere Visual 26, exhibited high importance weights. Moreover, left hemisphere regions such as Left Hemisphere Limbic Temporal Pole 6 and Left Hemisphere Visual 27 were also significantly activated. The left hemisphere somatosensory-motor cortex, Left Hemisphere Somatomotor 17, showed notable activity. Figure 5 presents a visualization of the top 5 percent of the most influential ROIs during the 0-back task, accompanied by Table A1, which outlines the details of the brain regions.

**Figure 6:**
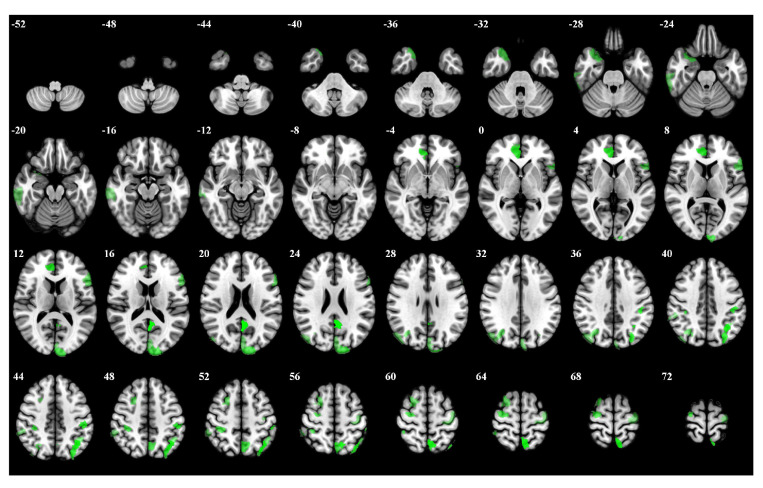
The top 5 percent of the most influential ROIs for 1-back, with higher opacity indicating higher influence weights.

In the 1-back task, participants were required to respond whenever the current stimulus matched the one presented immediately before it. Left hemisphere regions demonstrated prominent activation, including Left Hemisphere Default Precuneus Posterior Cingulate Cortex 3 and Left Hemisphere Dorsal Attention Posterior 15. Furthermore, left hemisphere regions such as Left Hemisphere Frontoparietal Control Parietal 4 and Left Hemisphere Frontoparietal Control Parietal 1 showed significant involvement. The top 5 percent of the most influential ROIs for the 1-back task is visualized in Figure 6, and Table A2 offers specific details on the brain regions.

**Figure 7:**
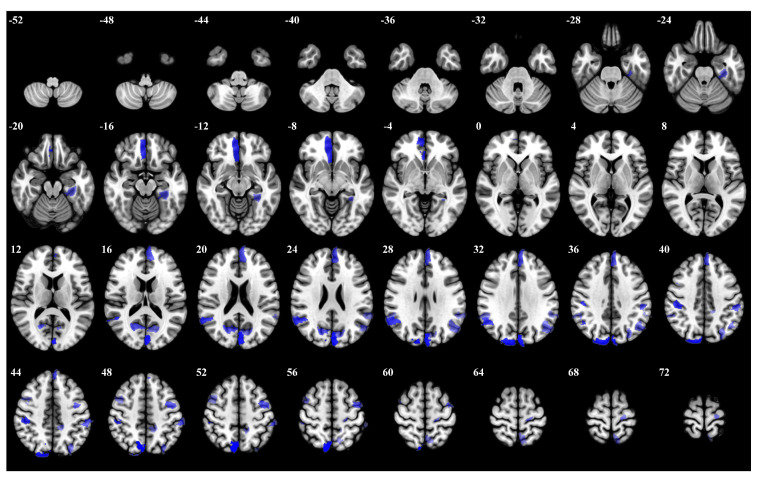
The top 5 percent of the most influential ROIs for 2-back, with higher opacity indicating higher influence weights.

In the 2-back task, participants had to respond whenever the current stimulus matched the one presented two stimuli back. The right hemisphere dorsal attention network exhibited substantial activation, particularly regions Right Hemisphere Dorsal Attention Posterior 15 and Right Hemisphere Dorsal Attention Posterior 9. Moreover, the bilateral visual cortex regions Left Hemisphere Visual 25 and Right Hemisphere Visual 29 showed significant activation. Figure 7 and Table A3 provide the details for the 2-back task, as above.

### 3.2. Comparative Experiments in Different Prior Knowledge Conditions

The previous experiment combined three N-back time series from subjects performing the same task throughout the experiment to classify three task types. However, the experiment design conducted these three tasks in a staggered order. Consequently, the same task varied in terms of prior knowledge, with differences in difficulty sequence, such as starting with easy and then transitioning to hard, or vice versa. To investigate these three types of working memory tasks in greater depth, we designed comparative experiments to classify the same difficulty of N-back tasks at different periods and to classify different difficulties of N-back tasks simultaneously.

Given that the sampling time for a single task is only 20 time points, we extended the experiment to 60 time points through replication. To validate the feasibility of the replication method, we conducted classification experiments on the dataset obtained through replication expansion and compared the classification results with the original dataset. Following simple parameter adjustments, the classification accuracy reached 67.76%. For comparison, the highest classification accuracy achieved in a single period was 67.10%. Therefore, it can be inferred that the replication expansion method effectively maintains the classification performance within the same period.

We extracted data corresponding to 0-back, 1-back, and 2-back tasks and conducted three separate classification experiments to assess their ability to distinguish among different prior situations. Table 2 shows the results of the classification task.

The classification results indicate a gradual decrease in accuracy as the task difficulty increased. This observation suggests that brain activity in regions associated with more challenging tasks exhibited greater similarity and was less influenced by prior knowledge. Conversely, easier tasks were more susceptible to sequencing and level of difficulty.

Next, we proceeded to classify the three tasks at different stages. The classification results are shown in Table 3.

It can be observed that the initial phase model exhibits better classification performance, characterized by a higher discriminability of activated brain regions, whereas the subsequent two phases show similar classification results. It is conjectured that participants lacked proficiency in the classification task at the beginning, indicating a learning curve. Participants likely became more adept at capitalizing on repeated information as the task progressed, mitigating task difficulty. Consequently, the activation of brain regions began to decline and stabilize.

## 4. Discussion

Based on the BABRI cohort, this study innovatively adopted a novel transformer structure that effectively captures local-to-global representations of time series to perform detection tasks based on fMRI scans of the N-back task. The architecture learned latent representations of fMRI data via a novel fused window attention mechanism that incorporates long-range context with linear complexity regarding scan length. Detection was then performed based on learned high-level classification tokens regularized across time windows. We then used a matched explanatory technique to calculate the weights of activated brain regions to obtain each brain region’s contribution to the task. In the 0-back task, the right hemisphere visual cortex exhibited high importance weights. This suggests its crucial involvement in visual processing and discrimination of the target stimulus, possibly reflecting the visual encoding and identification of the presented number. In the left hemisphere, the involvement of the limbic temporal pole might indicate emotional processing or memory retrieval associated with the presented stimuli [40,41]. Meanwhile, the visual cortex likely contributed to visual perception and recognition. The engagement of motor responses reflects the participants’ manual responses to the target stimuli. In the 1-back task, the activation of the default precuneus posterior cingulate cortex in the left hemisphere, associated with the default mode network (DMN), suggests involvement in maintaining attentional focus and cognitive control during the task [42,43,44,45]. Dorsal attention posterior in the left hemisphere, a dorsal attention network (DAN) component, likely played a role in sustaining attention and monitoring for target stimuli [46,47]. Part of the parietal cortex, including the control parietal in the left hemisphere involved in attentional control, likely facilitated the comparison and matching processes required in the task [48,49,50]. In the 2-back task, the substantial activation of the dorsal attention network in the right hemisphere suggests their role in maintaining attentional resources and updating working memory representations across trials in the task [51,52]. The participation and activation of bilateral visual cortex regions indicate their involvement in visual processing and encoding stimuli, supporting the participants’ recognition and discrimination of the target numbers.

The observed activation patterns across the three tasks underscore the distributed nature of cognitive processing, with different brain regions contributing to various cognitive demands. The involvement of visual cortex regions in all tasks highlights the fundamental role of visual perception and discrimination in task performance. Furthermore, the engagement of attentional networks, including the default mode and dorsal attention networks, suggests the importance of attentional control and cognitive monitoring across tasks. These networks likely play a crucial role in regulating attentional resources and maintaining task-relevant information in working memory. These findings are consistent with the existing literature on the correlates of N-back tasks. In comparison to previous studies, we assigned precise importance weights to all brain regions, enabling an evaluation of the entire brain’s contributions rather than limiting the analysis to a few prominent regions. This perspective provides more detailed insights into the mechanisms underlying cognitive control and working memory processing.

In the comparative experiment, the observed decrease in classification accuracy with increasing task difficulty suggests a nuanced relationship between task complexity and brain activation. As tasks become more challenging, neural resources may be recruited more uniformly, reflecting adaptive strategies to cope with increased cognitive demands. Furthermore, the differential impact of prior knowledge on task classification highlights the role of cognitive factors in shaping brain activation patterns. Easier tasks are more susceptible to the influence of previous knowledge, indicating a potential reliance on familiar strategies or mental models. In contrast, more difficult tasks exhibit greater consistency in brain activation, possibly reflecting a higher reliance on core cognitive processes unaffected by prior experiences. The temporal dynamics of brain activation across task stages underscore the importance of considering learning effects in neuroimaging studies. The superior performance of the initial phase model suggests a period of exploration and adaptation, where participants familiarize themselves with task requirements. Subsequent phases show stabilization in brain activation, indicating optimized cognitive processing and reduced reliance on novel strategies.

However, our study primarily focuses on individuals above 50, which may not accurately reflect brain activation patterns in younger individuals or those with different health statuses due to age-related changes in brain structure and function and varying life experiences. Moreover, excluding participants with neurological or psychiatric disorders limits our understanding of how these conditions affect neural correlates of cognitive tasks. Future research should include a broader range of ages and clinical backgrounds to enhance the generalizability of findings and uncover unique patterns associated with diverse populations.

## 5. Conclusions

This study employs a novel deep learning technique to investigate the spatiotemporal brain activation patterns during working memory tasks. The classification experiments identified the critical brain regions contributing to the cognitive task, providing new insights into the neural mechanisms and producing more accurate and comprehensive results. Additionally, comparative experiments revealed differences in brain activation patterns under varying task difficulty and prior knowledge conditions. These findings highlight the adaptive strategies of neural resources in response to increased cognitive demands and underscore the role of cognitive factors in shaping brain activation patterns. The results also validate the optimization of brain region stability in cognitive strategies following adaptation.

## Figures and Tables

**Table 1:** The hyperparameter adjustment process in the classification task. The results demonstrate that a receptive field with more layers can increase classification accuracy under a larger dynamic length condition. Bold values indicate the parameter values and results that achieve the highest accuracy under the same conditions.

*D*	*N*	*W*	α	Accuracy	ROC
20	4	20	0.4	57.12%	74.94%
30	4	20	0.4	58.17%	75.66%
40	4	20	0.4	61.96%	80.30%
**50**	4	20	0.4	**63.66%**	**83.50%**
50	4	10	0.4	66.41%	84.93%
50	4	**5**	0.4	**70.72%**	**86.93%**
50	4	5	**0.6**	**71.37%**	86.91%
50	**6**	5	0.6	**72.29%**	**88.00%**
50	6	5	**0.8**	**73.07%**	**88.46%**
50	**12**	5	0.8	**73.86%**	**89.02%**

**Table 2:** Comparative experiment of the same task under different prior knowledge conditions.

Task Type	Accuracy	ROC
0-back	64.31%	82.62%
1-back	56.07%	75.21%
2-back	41.05%	58.80%

**Table 3:** Comparative experiment of the task classification at different time stages.

Task Stage	Accuracy	ROC
012	67.58%	84.46%
120	63.53%	82.22%
201	63.79%	82.32%

## Data Availability

Due to privacy and ethical restrictions, the data used in this study are not publicly available. The analysis scripts and data supporting the findings of this study can be obtained from the corresponding author upon reasonable request.

## Appendix A

**Table A1:** The specific information of the top 5 percent of the most influential ROIs for 0-back.

Rank	Brain Regions	Side	Centroid Coordinates (R A S)	Importance Weight
1	Visual 19	R	9 −74 9	0.315
2	Visual 26	R	27 −87 21	0.314
3	Limbic Temporal Pole 6	L	−40 −21 −27	0.274
4	Visual 27	L	−12 −71 20	0.272
5	Somatomotor 17	L	−51 −7 43	0.271
6	Frontoparietal Control Cingulate 1	R	6 −26 28	0.253
7	Salience Ventromedial Attention Medial 4	R	12 −34 43	0.247
8	Visual 5	L	−23 −73 −10	0.238
9	Visual 18	R	35 −89 2	0.238
10	Dorsal Attention Posterior 16	L	−20 −57 66	0.234
11	Somatomotor 21	R	52 −13 49	0.231
12	Somatomotor 10	R	41 −29 18	0.228
13	Frontoparietal Control Lateral Prefrontal Cortex 8	R	48 18 23	0.228
14	Default Temporal 5	L	−53 6 −11	0.226
15	Default Precuneus Posterior Cingulate Cortex 2	L	−13 −61 19	0.225
16	Default Dorsal Prefrontal cortex an Medial Prefrontal Cortex 13	R	12 20 63	0.217
17	Visual 28	L	−32 −84 27	0.216
18	Frontoparietal Control Parietal 5	R	54 −33 51	0.204
19	Somatomotor 3	R	53 −14 6	0.203
20	Default Dorsal Prefrontal cortex and Medial Prefrontal Cortex 12	R	12 −55 15	0.203

**Table A2:** The specific information of the top 5 percent of the most influential ROIs for 1-back.

Rank	Brain Regions	Side	Centroid Coordinates (R A S)	Importance Weight
1	Default Precuneus Posterior Cingulate Cortex 3	L	−4 −53 20	0.246
2	Frontoparietal Control Parietal 4	L	−35 −62 48	0.241
3	Dorsal Attention Posterior 15	L	−7 −59 63	0.237
4	Limbic Temporal Pole 2	L	7 42 4	0.216
5	Dorsal Attention Posterior 8	L	−46 −29 44	0.212
6	Frontoparietal Control Parietal 1	L	−29 −74 42	0.211
7	Somatomotor 31	R	29 −11 65	0.206
8	Visual 24	L	−11 −97 17	0.195
9	Frontoparietal Control Precuneus 2	L	−5 −64 52	0.187
10	Dorsal Attention Posterior 13	R	35 −36 51	0.181
11	Limbic Temporal Pole 5	R	29 12 −30	0.176
12	Frontoparietal Control Parietal 5	L	−42 −52 49	0.174
13	Dorsal Attention Posterior 5	R	32 −66 35	0.171
14	Default Prefrontal Cortex 10	L	−53 19 11	0.169
15	Frontoparietal Control Lateral Prefrontal Cortex 15	R	24 10 58	0.165
16	Frontoparietal Control Temporal 1	R	62 −28 −20	0.163
17	Somatomotor 26	L	−36 −19 65	0.161
18	Visual 25	L	−3 −84 24	0.159
19	Dorsal Attention Posterior 4	R	45 −75 31	0.157
20	Frontoparietal Control Parietal 2	R	56 −41 48	0.153

**Table A3:** The specific information of the top 5 percent of the most influential ROIs for 2-back.

Rank	Brain Regions	Side	Centroid Coordinates (R A S)	Importance Weight
1	Dorsal Attention Posterior 15	R	8 −71 53	0.287
2	Visual 25	L	−3 −84 24	0.285
3	Visual 29	R	16 −87 36	0.256
4	Visual 19	L	5 41 −11	0.255
5	Dorsal Attention Posterior 9	R	45 −28 42	0.252
6	Default Parietal 3	R	53 −53 26	0.241
7	Visual 24	R	16 −66 19	0.237
8	Dorsal Attention Frontal Eye Fields 1	L	−40 −3 51	0.227
9	Default Prefrontal Cortex 13	L	−4 51 28	0.223
10	Dorsal Attention Posterior 6	L	−55 −32 45	0.218
11	Frontoparietal Control Parietal 1	L	−29 −74 42	0.214
12	Visual 2	L	−30 −33 −18	0.203
13	Default Parietal 4	L	−47 −64 31	0.200
14	Visual 26	L	−12 −71 20	0.195
15	Somatomotor 31	L	−19 −24 67	0.185
16	Salience Ventromedial Attention Parietal Operculum 2	L	−58 −44 27	0.183
17	Default Parietal 4	R	55 −45 33	0.182
18	Salience Ventromedial Attention Medial 5	L	−13 −41 47	0.180
19	Dorsal Attention Posterior 15	L	−7 −59 63	0.177
20	Frontoparietal Control Lateral Prefrontal Cortex 13	R	43 7 51	0.176
